# Ag- and Zn-clinoptilolite: a comparison of their *in-vitro* antibacterial activity against *Helicobacter pylori*

**DOI:** 10.1007/s10653-025-02868-0

**Published:** 2025-11-04

**Authors:** Guido Cerri, Antonio Brundu, Claudia C. Juliano, Mauro Farina

**Affiliations:** 1 Dipartimento di Architettura, Design e Urbanistica - GeoMaterials Lab, Università degli Studi di Sassari, Via Piandanna 4, 07100 Sassari, Italy; 2 Dipartimento di Medicina, Chirurgia e Farmacia, Università degli Studi di Sassari, Via Muroni 23, 07100 Sassari, Italy; 3 Azienda Socio Sanitaria Locale-ASL Nuoro, Farmacia Territoriale, Via Amerigo Demurtas 1, 08100 Nuoro, Italy

**Keywords:** Zeolite, *Helicobacter pylori*, Silver, Zinc, Antimicrobial resistance, Cation exchange

## Abstract

*Helicobacter pylori* is a bacterium recognized as the most frequent cause of chronic gastritis and is classified as carcinogen by the World Health Organization. For the eradication of *H. pylori*, the use of silver and zinc was studied due to their antibacterial properties. The use of clinoptilolite was also considered, since ammonium is essential for the survival of the bacterium in the stomach and clinoptilolite is acid resistant and selective toward NH_4_^+^. This research compared the antibacterial activity against *H. pylori* of Ag- and Zn-clinoptilolite through *in-vitro* tests, aiming to exploit the zeolite simultaneously as a metal supplier and as an ammonium sequestrant. A powder containing approximately 90% of clinoptilolite was used to prepare, by cation exchange, two materials based on Zn-clinoptilolite (FA-Zn) and Ag-clinoptilolite (FA-Ag), containing 2.12 meq/g of Zn^2+^ and 2.28 meq/g of Ag^+^, respectively. In the agar cup test, both materials evinced a direct and linear relationship between their concentration and the width of the inhibition zones, but FA-Ag allowed the onset of the development of an inhibition halo already at a concentration of 12.5 mg/mL, whereas FA-Zn required 25 mg/mL to get the same result. The Minimum Inhibitory Concentration of FA-Ag resulted eight time lower than that of FA-Zn (0.5 and 4.0 mg/mL, respectively). Tests showed superior antimicrobial activity of FA-Ag, but it should be considered that Ag-clinoptilolite has some disadvantages compared to Zn-clinoptilolite, such as higher toxicity, higher cost, and instability to light.

**Graphical Abstract:**

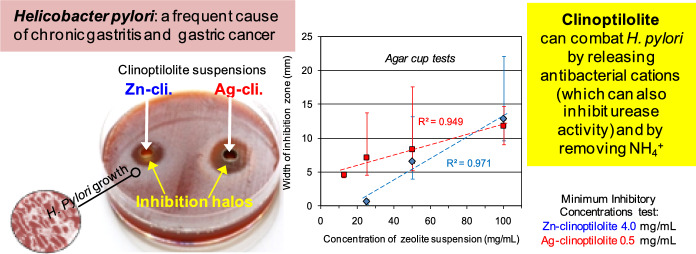

## Introduction

### *Helicobacter pylori* (*H. pylori*)

*H. pylori* is a Gram-negative, spiral-shaped microaerophilic bacterium recognized as the most frequent cause of chronic gastritis (Malfertheiner et al., 2023). Moreover, *H. pylori* infection is an important causal factor of gastric cancer, gastric ulcer and duodenal ulcer (Malfertheiner et al., 2023), and this bacterium is classified as class I carcinogen by the World Health Organization (Friedrich & Gerhard, 2023). In adults, the global prevalence of *H. pylori* has declined from 52.6% before 1990 to 43.9% during 2015 through 2022, but in the latter span of time was as still as high as 35.1% in children and adolescents (Chen et al., 2024).

### Therapies for the eradication of *H. pylori* and related issues

Therapies for the eradication of *H. pylori* associate two or three antibiotics (the most used are clarithromycin, amoxicillin, metronidazole, and levofloxacin) with an acidity suppressant, which is essential to optimize the stability, bioavailability and efficacy of the antibiotics in the gastric environment (Friedrich & Gerhard, 2023; Malfertheiner et al., 2023); some treatments include bismuth salts instead of a third antibiotic (Friedrich & Gerhard, 2023; Malfertheiner et al., 2023). Unfortunately, eradication therapies fail in up to 40% of cases, mainly due to the development of antibiotic-resistant bacterial strains (Fonseca et al., 2024). Of note, *H. pylori* is among the 16 bacteria of greatest concern in terms of drug resistance (Fonseca et al., 2024). Antibiotic resistance rates of *H. pylori* show significant variations, for example the rate of clarithromycin usually ranges from 15 to 30% worldwide (Malfertheiner et al., 2023), but reaches 81.3% in Vietnam (Tran et al., 2024). Antibiotic resistance can vary greatly even within the same country, as in China, where resistance rates of *H. pylori* to metronidazole and levofloxacin, among the highest in the world, are 77 and 33% respectively (Yin et al., 2023), although in Shanghai they are 45.8 and 2.5% respectively (Li et al., 2024).

### Studies on the possible use of silver and zinc in therapies for the eradication of *H. pylori*

Several approaches were explored to address the problem of *H. pylori* drug resistance, including the use of metals such as silver and zinc, exploited in different forms and associated with a variety of compounds (Bu et al., 2024; Fan et al., 2022; Fonseca et al., 2024; Lopes et al., 2014; Pop et al., 2022; Yin et al., 2023). These metals were selected for their inherent antibacterial properties (Slavin et al., 2017), historically exploited in multiple applications, from the production of burn wound dressing to the preparation of antibacterial soaps (Noor et al., 2022; Nowak et al., 2022). Another aspect worth considering is that zinc and silver can inhibit the activity of urease, an enzyme produced by *H. pylori* to catalyze the hydrolysis of urea, which is a fundamental process for the survival of the bacterium in an acidic environment (Fonseca et al., 2024; Yin et al., 2023).

### Previous studies on the possible use of zeolites in therapies for *H. pylori* eradication

A large number of scientific publications discuss the use of metal-exchanged zeolites as an antimicrobial agent (Demirici et al. 2014; Milenkovic et al., 2017; Dutta & Wang, 2019; Gomes et al., 2020; Alobaid et al., 2025; Guedes et al., 2025; Tabesh et al., 2025), but only a few experiments were performed with *H. pylori*, testing: i) Na- and NH_4_-clinoptilolite (Farina et al., 2019); ii) a powdered rock composed of clinoptilolite and mordenite, which was tested both unexchanged and prepared in Na and Zn form (Cerri et al., 2021); iii) zeolite-like materials (albeit not zeolites *stricto *sensu; Chen et al., 2023) such as metal–organic frameworks (MOFs) containing zinc (Bu et al., 2024). The experiments with clinoptilolite (± mordenite) represent an attempt to exploit zeolite’s properties, such as cation exchange capacity and selectivity towards NH_4_^+^, against *H. pylori* (Cerri et al., 2021; Farina et al., 2019). In fact, to survive in the acidic gastric environment (pH < 2.0) the bacterium hydrolyzes the urea present in the stomach producing CO_2_ and NH_3_ and, due to H^+^ presence, ammonia is converted to ammonium, thus determining a local raise of pH (to ≈6.1) around the bacterium (Malfertheiner et al., 2023). Removing ammonium ions could be a way to weaken *H. pylori*’s protective layer (Fonseca et al., 2024). *In-vitro* tests demonstrated that Na-clinoptilolite inhibits *H. pylori* growth, and this was attributed to the ability to subtract NH_4_^+^ by ion exchange (Farina et al., 2019). In fact, the same zeolite in ammonium form did not evidence antibacterial activity, as unable to remove NH_4_^+^ (Farina et al., 2019). On the other hand, the concentrations of zeolite necessary to inhibit the growth of *H. pylori* are too high to be of practical use (Cerri et al., 2021; Farina et al., 2019), particularly regarding the material tested in its natural (poly)cation composition, due to the lower efficiency of the ion exchange process with ammonium (Cerri et al., 2021). Interestingly, Na-clinoptilolite showed synergy with amoxicillin against *H. pylori*, as revealed by disc diffusion tests (Farina et al., 2019). Finally, the experiments performed with the material composed mainly (≈70%) of clinoptilolite and mordenite in Zn-form showed a Minimum Inhibitory Concentration (MIC) 7.5 times lower than the corresponding sodium counterpart (Cerri et al., 2021).

### Aim of the work

The previous experiments demonstrated that the activity of a zeolite-based system against *H. pylori* can be enhanced by coupling ammonium subtraction with the simultaneous release of an antimicrobial metal ion such as zinc (Cerri et al., 2021). The purpose of this work was to compare, through *in-vitro* tests and using a material containing 90% of clinoptilolite, the antibacterial activity against *H. pylori* of Ag- and Zn-clinoptilolite, in the hope of obtaining potentially useful materials for developing adjuvants for *H. pylori* eradication therapies.

## Experimental

### Starting material

The zeolite used in this work is clinoptilolite, the most common natural zeolite, defined as the mineral series with heulandite (HEU) framework topology and Si/Al ≥ 4 (Cerri & Brundu, 2022). Due to its resistance in an acidic environment, in oral administration clinoptilolite is preferred to other more aluminous natural zeolites (Milić et al., 2014). To get a meaningful comparison, silver and zinc clinoptilolite were prepared starting from the same material, as this approach prevents factors relevant in the release of antimicrobial metal ions (such as zeolite content, structure topology, Si/Al ratio, pore size, and specific surface area - Cerrillo et al., 2017; Dutta & Wang, 2019) from influencing the comparison. The material employed for the research comes from the lot previously prepared by Cerri et al. (2016) for the development of pharmaceuticals. This material was obtained by subjecting to a beneficiation process a clinoptilolite-rich rock collected in Sardinia (Italy), obtaining a powder with a grain size between 0.4 to 80 μm and modal diameter of 10.83 ± 0.35 μm, composed (in weight) by 90.2 ± 2.0% of clinoptilolite, 0.4 ± 0.1% of quartz, 1.2 ± 0.2% of biotite, 3.2 ± 0.3% of feldspars, 1.2 ± 0.2% of opal-CT and 3.8 ± 1.0% of amorphous (Cerri et al., 2016).

### Preparation of Ag- and Zn-clinoptilolite

To increase the efficiency of the ion exchanges to be performed with zinc and silver, the powder was initially submitted to Na-exchange using a 1 M NaCl solution (VWR International, European Pharmacopoeia (Ph. Eur.) grade; purity 99.9%). Setting a solid/liquid ratio of 30 g/L, 10 exchange cycles (each of 2 h) were performed at 65 °C under continuous stirring (300 rpm—IKA RCT basic magnetic stirrer). The powder was recovered by centrifugation (Hettich Universal 320 centrifuge) at the end of each cycle, then repeating the ion exchange process. After the last cycle, the powder was washed with deionized water and recovered by centrifugation, repeating this procedure until no more chloride was detected in the elutes (test performed with AgNO_3_; Jeffrey et al., 1989), then the powder was dried overnight at 40 °C. To allow rehydration, the final material (labeled as FA-Na2) was left in a desiccator containing a saturated solution of Ca(NO_3_)_2_ for 24 h at 22 °C and 53 ± 2% of relative humidity.

To prepare Zn-clinoptilolite, FA-Na2 was contacted with a 0.5 M solution of ZnSO_4_∙7H_2_O (VWR International, Ph. Eur. grade, purity > 99%). The exchange procedure was the same as described to get FA-Na2, with the following differences: i) the duration of the first two exchange cycles was 1 h; ii) at the end of the last cycle, the powder (hereafter, FA-Zn) was washed 14 times with deionized water.

To prepare Ag-clinoptilolite, FA-Na2 was contacted with a 0.5 M solution of AgNO_3_ (VWR International, Ph. Eur. grade, purity > 99%). The exchange procedure was the same as described to get FA-Na2, with the following differences: i) during the process, dark containers were used to avoid reduction of Ag^+^ to Ag^0^ (Cerrillo et al., 2017); ii) 7 exchange cycles were performed; iii) the duration of the first two exchange cycles was 1 h; iv) at the end of the last cycle, the powder (hereafter, FA-Ag) was washed with deionized water until no more silver was detected in the elutes (Jeffrey et al., 1989).

### Chemical analyses

The samples FA-Na2, Fa-Zn, and Fa-Ag were analyzed at the Activation Laboratories Ltd (Actlabs—Ancaster, ON, Canada). Major elements were determined after lithium metaborate/tetraborate fusion of the sample through Inductive Coupled Mass Atomic Emission Spectrometry (ICP-AES), carried out with a Varian Vista 735 ICP. The content of zinc was measured by ICP Mass Spectrometry (ICP-MS; Perkin Elmer Sciex ELAN 9000) after sodium peroxide fusion. Silver was determined through Instrumental Neutron Activation Analysis (INAA). The Loss on Ignition (LoI) of the samples was measured in our laboratories by thermogravimetry (see Sect. "Thermal analyses").

### Thermal analyses

Thermogravimetric (TG) analyses were performed using a TA Instrument Q600 (CeSAR—Centro Servizi di Ateneo per la Ricerca, Sassari University). Samples of about 20 mg were heated to 900 °C using an alumina crucible and setting the following operating conditions: heating rate 10 °C/min; air flow 100 mL/min. The software TA-Universal Analysis 2000 V 4.5A was employed to evaluate the results.

### X-ray diffraction

X-ray diffraction (XRD) analyses were carried out using a diffractometer Bruker D2 Phaser. The instrumental parameters were as follows: CuKα radiation, voltage 30 kV, current 10 mA, LynxEye PSD detector with angular opening of 5°, 2θ range 6–70°, step size 0.020°, time per step of 2 s, sample spinner 30 rpm. Before measurement, the samples were micronized (Retsch MM400 mill, equipped with grinding media in ZrO_2_). The X-ray patterns were evaluated using the software EVA 14.2 (Bruker DIFFRAC^plus^) combined with the PDF-2 database (International Centre for Diffraction Data). The cell parameters of Ag- and Zn-clinoptilolite were determined by employing the Le Bail method and the software Bruker Topas 5.

### Agar cup test

All *in-vitro* tests were performed using the reference strain of *H. pylori* American Type Culture Collection (ATCC^®^) 43504™. For the culture of *H. pylori*, the instructions of Megraud and Lehours (2007) were followed. The agar cup (or agar well) diffusion method (Balouiri et al., 2016; Sonibare et al., 2011) was used to assess the susceptibility of the bacterium against Ag- and Zn-clinoptilolite. The test was performed by pouring 20 mL of Mueller Hinton Agar (Oxoid) supplemented with 5% of defibrinated horse blood (Oxoid) into a series of Petri dishes (Ø 80 mm). Afterwards, a suspension of *H. pylori* having a turbidity equivalent to a 4.0 McFarland standard, prepared using sterile saline (NaCl 9 g/L), was streaked on the solidified culture medium using a sterile swab, and two dishes were set aside to be used as control. Two cups, 7.9 mm in diameter, were excavated in the solidified agar contained in the other dishes, using the wider end of a pipette tip as an auger (Balouiri et al., 2016). In each plate, one cup was filled with 50 µL of a suspension (prepared using bi-distilled water) of Ag-clinoptilolite, and the second cup with the same volume of a suspension of Zn-clinoptilolite (Balouiri et al., 2016; Cerri et al., 2021). The zeolite materials were tested at concentrations of 12.5, 25, 50 and 100 mg/mL. Once the liquid into the cups was absorbed (≈ 20 min required), the dishes were incubated, upside down, at 37 °C for three days in microaerophilic conditions (85% N_2_, 5% O_2_, and 10% CO_2_—CampyGen™ sachets, Oxoid). The dishes were visually checked for bacterial growth after incubation. When present, the width of the inhibition halo (i.e., the area surrounding the cup where no bacterial growth occurred) was measured with a caliper. The width of the *H. pylori* growth inhibition zone is given by the difference between the diameter of the inhibition halo (Ø_IH_) and the diameter of the cup (Ø_C_) excavated in the agar.

### Minimum inhibitory concentration (MIC)

The agar medium, prepared as described in Sect. "Agar cup test", was poured into a set of Petri dishes (20 mL per plate). The zeolite powders were then dispersed into the still liquid culture medium by preparing, for both Ag- and Zn-clinoptilolite, a series of plates with zeolite concentration of 0.125, 0.25, 0.5, 1, 2, 4, 6, and 8 mg/mL, following the procedure described for the "agar dilution method" (Balouiri et al., 2016). Once the growth medium had solidified, two 3 μL spots of bacterial suspension (see Sect. "Agar cup test") were deposited on its surface (Balouiri et al., 2016; Cerri et al., 2021), then the dishes were incubated for 3 days in the microaerophilic conditions already described in the previous section. After incubation, the plates were visually inspected for bacterial growth to determine the MIC of Ag- and Zn-clinoptilolite, i.e., the lowest concentration of each zeolite form that inhibits the growth of *H. pylori*. The tests were performed in duplicate, and a couple of dishes without zeolite were used as control in each experiment.

## Results and discussion

Table 1 shows the chemical composition of the material achieved after the Na-, Zn- and Ag-exchange processes.

**Table 1:** Chemical composition (in wt.%) of Na-clinoptilolite (FA-Na2), Zn-clinoptilolite (FA-Zn) and Ag-clinoptilolite (FA-Ag)

	SiO_2_	Al_2_O_3_	Fe_2_O_3_	MnO	MgO	CaO	Na_2_O	K_2_O	TiO_2_	P_2_O_5_	ZnO	Ag_2_O	LoI	TOT
FA-Na2	64.51	12.53	0.69	< 0.01	0.50	0.33	6.00	0.40	0.19	0.05	–	–	14.61	99.82
FA-Zn	62.03	11.93	0.66	< 0.01	0.32	0.31	0.25	0.37	0.18	0.04	8.56	–	15.69	100.35
FA-Ag	50.50	9.27	0.44	< 0.01	0.20	0.21	0.12	0.23	0.13	0.04	–	26.64	12.13	99.96

**Fig. 1:**
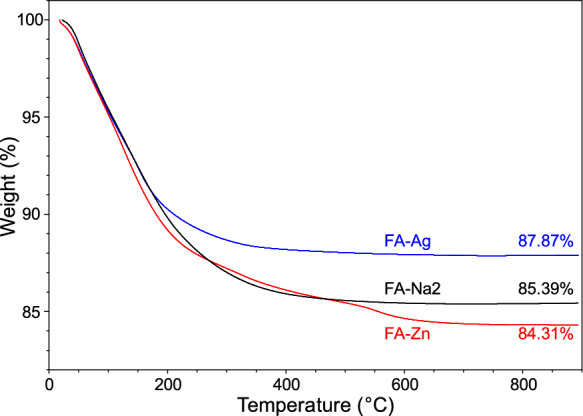
Thermogravimetric analyses of Na-clinoptilolite (FA-Na2), Zn-clinoptilolite (FA-Zn) and Ag-clinoptilolite (FA-Ag)

The two metallic forms present low amounts of Mg, Ca, Na and K which were not released during the exchange process mainly because they were contained in the residual non-zeolitic fraction (< 10%, see Sect. “Starting material”). The LoI values determined by TG (Fig. 1), show that the water content decreases from FA-Na2 to FA-Ag, whereas it increases from FA-Na2 to FA-Zn. In zeolites, H_2_O molecules can occupy the cation-free portion of microporosity (Esposito et al., 2015), hence, given the higher ionic radius of silver compared to sodium, the replacement of Na^+^ with Ag^+^ reduces the space available for water, while the substitution of two sodium cations with one Zn^2+^ leaves more space for H_2_O molecules, also due to the smaller ionic radius of zinc.

**Fig. 2:**
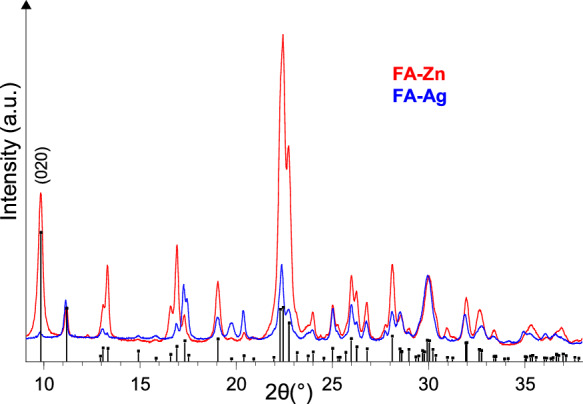
X-ray diffraction patterns of Zn-clinoptilolite (FA-Zn) and Ag-clinoptilolite (FA-Ag), detail in the 2θ range 9°–38°. Black bars: clinoptilolite (PDF N. 80–0464)

FA-Zn contains 2.12 meq/g of Zn^2+^, whereas the silver amount in FA-Ag corresponds to 2.28 meq/g of Ag^+^. It is possible that not all the silver contained in FA-Ag is present as exchangeable cation within the clinoptilolite channels, because a known drawback that may occur in preparing Ag-exchanged zeolites is the formation of silver clusters and/or of silver oxide, which can also result in a darkening of the material (Cerrillo et al., 2017, 2020; Concepción-Rosabal et al., 2005). Light and temperature influence the onset of these problems (Cerrillo et al., 2017; Concepción-Rosabal et al., 2005), that’s why the preparation of FA-Ag was done using dark containers and limiting the temperature to 65 °C during the exchange, and to 40 °C in the drying process. Despite these precautions, FA-Ag powder shows a light grey color, while FA-Zn is white like FA-Na2. However, the X-ray pattern of FA-Ag shows no peaks of metallic silver and/or silver oxides (Fig. 2), so these phases, if present, could be amorphous or, if crystalline, below the detection limit (Holder & Schaak, 2019). Figure 2 shows that the intensity of Ag-clinoptilolite peaks is generally lower than that of Zn-clinoptilolite (which, in turn, is weaker than that of Na-clinoptilolite (Cerri et al., 2021). According to Cerrillo et al. (2017), the higher the amount of silver in Ag- zeolites, the lower the intensity of the peaks, and Dimowa et al. (2011) showed the relationship between the increase in silver content and the decrease in intensity of the (020) peak in clinoptilolite (peak labeled in Fig. 2). The cell parameters of the Ag-clinoptilolite contained in FA-Ag (refined in the Space Group C2/*m*) are the following: *a* = 17.648(3) Å; *b* = 17.990(4) Å; *c* = 7.404(3) Å; *β* = 116.23(3)°; *V* = 2108(4) Å^3^. For Zn-clinoptilolite (refined in the Space Group C2/*m* as well) the values obtained are:* a* = 17.649(2) Å; *b* = 17.955(2) Å; *c* = 7.416(2) Å; *β* = 116.25(3)°; *V* = 2108(5) Å^3^. The cell parameters of the two zeolites are in good agreement with those reported in the literature for Ag- (Dimowa et al., 2011) and Zn-clinoptilolite (Dimowa et al., 2015).

**Fig. 3:**
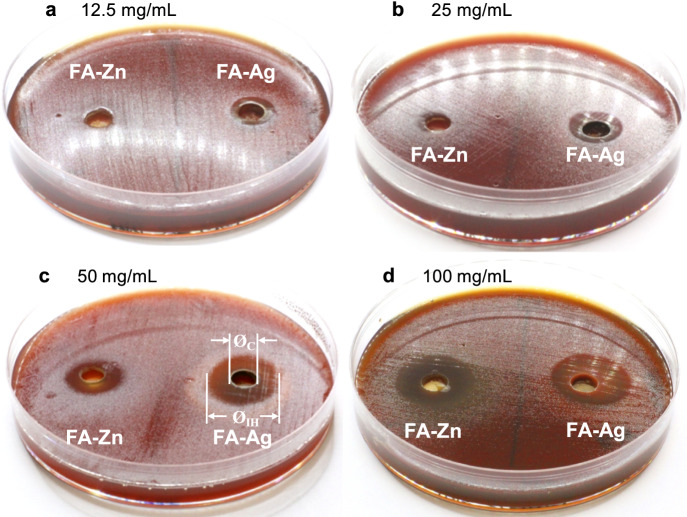
Agar cup test—Suspensions of Zn-clinoptilolite (FA-Zn) and Ag-clinoptilolite (FA-Ag) poured into the cups at the concentrations indicated. The width of the *H. pylori* growth inhibition zone is given by the difference between the diameter of the inhibition halo (Ø_IH_) and the diameter of the cup (Ø_C_) excavated in the agar

Figure 3 collects four representative images of agar cup tests performed using different concentrations of FA-Ag and FA-Zn, and also shows how the widths of *H. pylori* growth inhibition reported in Tables 2 and 3 were obtained. FA-Ag was able to inhibit bacterial growth already at a concentration of 12.5 mg/mL (Table 2), unlike FA-Zn which started to show weak antimicrobial activity against *H. pylori* only at 25 mg/mL (Table 3). Note that, when compared at the same concentrations, FA-Zn shows slightly wider inhibition halos than the previously tested material composed of Zn-exchanged clinoptilolite + mordenite (see M-Zn in Cerri et al., 2021), probably due to a higher zinc content achieved thanks to the higher percentage of zeolite (≈ 90% of clinoptilolite in FA-Zn vs. ≈ 70% of clinoptilolite + mordenite in M-Zn (Cerri et al., 2021)).

**Table 2:** Results of the agar cup test performed with Ag-clinoptilolite (FA-Ag)

FA-Ag concentration (mg/mL)	Width of inhibition zone (mm)				N. of tests done
	min	max	*average*	*st. dev*	
12.5	4.2	4.8	*4.6*	*0.1*	4
25	4.6	13.8	*7.2*	*2.9*	10
50	5.2	17.6	*8.4*	*3.0*	10
100	9.1	14.7	*11.9*	*1.9*	10

**Table 3:** Results of the agar cup test performed with Zn-clinoptilolite (FA-Zn)

FA-Zn concentration (mg/mL)	Width of inhibition zone (mm)				N. of tests done
	min	max	*average*	*st. dev*	
12.5	0.0	0.0			4
25	0.0	4.2	*0.7*	*1.2*	10
50	2.6	10.9	*6.6*	*2.3*	10
100	10.9	14.2	*12.9*	*1.2*	10

In general, the width of the inhibition halos of FA-Ag is wider than that of FA-Zn at the same concentration (Fig. 3), except at 100 mg/mL, when they reach similar average values (Tables 2 and 3). Both materials show significant variability in the extent of the halos, although the oscillations are slightly larger in FA-Ag (compare the standard deviation values in Tables 2 and 3).

**Fig. 4:**
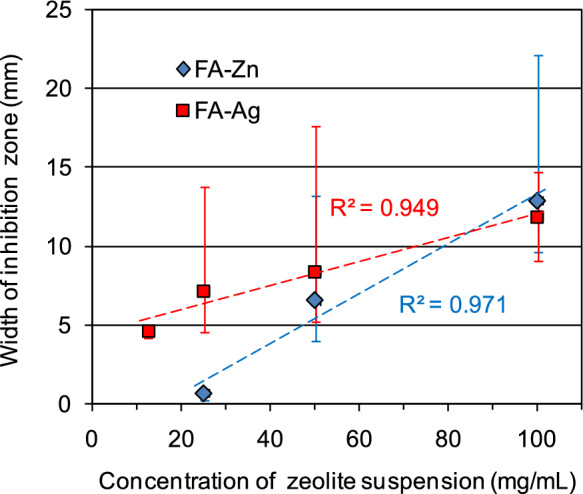
Relationship between the width of the inhibition halo and the concentration of the suspension of Zn-clinoptilolite (FA-Zn) and Ag-clinoptilolite (FA-Ag)

As clearly highlighted in Fig. 4, there is a linear relationship between the concentration of the two clinoptilolite-based materials and the width of the *H. pylori* growth inhibition zone. In particular, both FA-Ag and FA-Zn show high correlation coefficients (R^2^ 0.949 and 0.971, respectively).

The outcomes of the MIC determination are consistent with the agar cup test results in indicating the superior ability of FA-Ag to inhibit the growth of *H. pylori* compared to FA-Zn. The data in Table 4 show that, as far FA-Ag, inhibition occurred at a concentration of 0.5 mg/mL, whereas in the case of FA-Zn a concentration of 2.0 mg/mL still allowed stunted bacterial growth and inhibition was achieved only at 4.0 mg/mL. The results of the MIC obtained with FA-Zn are consistent with those reported by Cerri et al. (2021) testing the material composed of clinoptilolite + mordenite exchanged with Zn^2+^.

**Table 4:** Determination of the Minimum Inhibitory Concentration (MIC) of Ag-clinoptilolite (FA-Ag) and Zn-clinoptilolite (FA-Zn) on the growth of *H. pylori*

Sample	Zeolite concentration in the growth medium (mg/mL)							
	0.125	0.25	0.5	1.0	2.0	4.0	6.0	8.0
FA-Ag	n	n	i	i	i	i	i	i
FA-Zn	n	n	n	n	s	i	i	i

n—normal growth; s—stunted growth; i—inhibited growth

Experiments conducted on 34 clinical strains of *H. pylori* showed that the MIC of silver nitrate ranged from 16 to 64 μg/mL (Amin et al., 2012), corresponding to 10.2 to 40.6 μg/mL of Ag^+^, respectively. The MIC of FA-Ag is equivalent to a silver ion content of 124 μg/mL, a value three times higher than the highest MICs reported for the above-mentioned clinical strains (Amin et al., 2012). On the other hand, it should be emphasized that we tested a different bacterial strain and, more importantly, in our case Ag^+^ must be released through ion exchange from a solid (clinoptilolite), whereas in a solution of AgNO_3_ all silver cations are immediately available. When comparing the bactericidal effects of different Ag-bearing materials, Lalueza et al. (2011) emphasized that the ease with which silver ions are available plays a crucial role regarding the biocidal effectiveness. The issues and considerations discussed above can be extended to FA-Zn, indeed Fan et al. (2022) reported that the MIC of zinc chloride against a reference strain of *H. pylori* (*H. pylori* 26695) is 105 µg/ml, corresponding to 50.4 µg/ml of Zn^2+^, whereas in terms of zinc ions content the MIC of FA-Zn corresponds to 275 µg/ml of Zn^2+^. Experiments conducted by Rainsford et al. (1997) on a total of 26 clinical and standard strains of *H. pylori* show how the concentration of zinc capable of influencing bacterial growth can vary even using a highly soluble zinc salt such as ZnSO_4_.

The mechanisms by which silver and zinc can kill *H. pylori* include damaging bacterial cell wall, interfering with essential enzymes, generating reactive oxygen species (ROS), and disrupting DNA (Fonseca et al., 2024; Pop et al., 2022; Slavin et al., 2017; Yin et al., 2023). Metal cations such as Ag^+^ and Zn^2+^ can be adsorbed onto the negatively charged surface of Gram-negative bacteria such as *H. pylori*, leading to their possible penetration into the cell membrane which then causes intracellular damage (Pop et al., 2022; Slavin et al., 2017; Yin et al., 2023). Furthermore, zinc and silver are able to inhibit the activity of urease, the enzyme essential for the survival of *H. pylori* in the acidic gastric environment (Fonseca et al., 2024; Yin et al., 2023), which would constitute another advantage offered by the use of Ag- or Zn-clinoptilolite, since it can act as an efficient supplier of these ions. Note that inhibition of urease activity is considered a promising therapeutic way to fight *H. pylori* (Malfertheiner et al., 2023; Zhao et al., 2024). Urease is a Ni-dependent enzyme (Kumar et al., 2022; Maroney & Ciurli, 2021), and inhibition of its activity by Ag^+^ or Zn^2+^ has been related to their interference with nickel cations (Fonseca et al., 2024; Yin et al., 2023). The removal of Ni^2+^ could be another mechanism to fight *H. pylori* because the survival of the bacterium relies on a significant supply of nickel (Kumar et al., 2022; Maroney & Ciurli, 2021). Clinoptilolite can effectively remove Ni^2+^ when sodium and nickel are the only cations present in the solution (Biblioteca et al., 2023), but the uptake of Ni^2+^ is strongly reduced in presence of other cations due to the low selectivity of clinoptilolite towards Ni^2+^ (Oter & Akcay, 2007). Conversely, being extremely selective towards NH_4_^+^, clinoptilolite can remove ammonium ions surrounding *H. pylori* (Cerri et al., 2021; Farina et al., 2019), thus weakening the bacterium’s defenses against the acidic gastric environment. Destruction of the protective cloud of ammonium ions is another method to combat *H. pylori* (Fonseca et al., 2024).

Both literature data and our experimental results indicate that silver has a superior antimicrobial activity to zinc against *H. pylori*, but it should be considered that the therapeutic action must occur in the stomach. The gastric environment presents more favorable conditions for the release of zinc and silver cations from zeolite than the conditions of laboratory experiments with culture media, however the HCl contained in the stomach would heavily reduce the amount of bioavailable Ag^+^ due to the precipitation of AgCl (Lalueza et al., 2011), while ZnCl_2_, being very soluble, would not be formed (Fodor & Szűcs, 2023). Moreover, the issue of the potential short- and long-term toxicity of silver-bearing compounds in humans is an aspect worthy of consideration (Anfray et al., 2017; De Mori et al., 2020; Mikhailova, 2020; Padhye et al., 2023), while those containing zinc raise less concern (Bu et al., 2024; Lopes et al., 2014; Yin et al., 2023), due to the lower toxicity of Zn compared to Ag. In this regard, it should be noted that, according to the U.S. Environmental Protection Agency, as far oral administration the lowest-observed-adverse-effect level (LOAEL) for zinc is 0.91 mg/kg·day (U.S. Environmental Protection Agency, 2005), while for silver it is 1.4 × 10^–2^ mg/kg·day (U.S. Environmental Protection Agency, 1991). These data, referring to a body weight of 70 kg, correspond to 63.7 mg/day of zinc and 0.98 mg/day of silver. Another issue to consider when designing metal-bearing drugs for *H. pylori* eradication is the higher cost of silver compared to zinc (Yin et al., 2023). For example, the price per gram of the silver nitrate used to prepare FA-Ag is two order of magnitude higher than that of the zinc sulphate employed to realize FA-Zn. Finally, unlike Zn-clinoptilolite, Ag-clinoptilolite must be prepared and stored in the dark, as it tends to darken when exposed to light (Cerrillo et al., 2017, 2020; Concepción-Rosabal et al., 2005; Lalueza et al., 2011). The darkening would not only affect the color of the material but, by modifying the oxidation state of silver, also the kinetics of its release (Lalueza et al., 2011). Table 5 summarizes the advantages and disadvantages of Ag-clinoptilolite and Zn-clinoptilolite.

**Table 5:** Summary of the advantages and disadvantages of the two metallic forms of zeolite

	Ag-clinoptilolite	Zn-clinoptilolite
MIC against *H. pylori*	0.5 mg/mL	4.0 mg/mL
Cost	High	Low
Toxicity	High	Low
Stability (to light)	Unstable	Stable

## Conclusion

The cation exchange capacity of the two materials, prepared from the same powder containing approximately 90% clinoptilolite, was basically fully exploited, reaching 2.12 meq/g of Zn^2+^ and 2.28 meq/g of Ag^+^, respectively. The experiments performed *in-vitro* showed the superior antimicrobial activity against *H. pylori* of Ag-clinoptilolite compared to Zn-clinoptilolite. In the agar cup test, both materials evinced a direct and linear relationship between their concentration and the width of the inhibition zones, but Ag-clinoptilolite allowed the onset of the development of an inhibition halo already at a concentration of 12.5 mg/mL, whereas 25 mg/mL of Zn-clinoptilolite were required to obtain the same result. Moreover, the MIC of the zeolite in silver form resulted eight time lower than that of Zn-clinoptilolite (0.5 and 4.0 mg/mL, respectively). The silver and zinc zeolite forms prepared for the present research can release Ag^+^ or Zn^2+^ which, through different mechanisms, damage *H. pylori* and inhibit the activity of the enzyme urease (Fonseca et al., 2024; Pop et al., 2022; Slavin et al., 2017; Yin et al., 2023), essential for the survival of the bacterium in the stomach. Furthermore, by virtue of the high selectivity of clinoptilolite toward NH_4_^+^, the zeolite is able to reduce the ammonium layer surrounding *H. pylori* (Cerri et al., 2021; Farina et al., 2019), whose function is to protect the bacterium from the acidic gastric environment. Finally, clinoptilolite has antidiarrheal properties (Hrenović & Rajić, 2025; Langbein et al., 2019), and these may be useful considering that antibiotics used for the eradication of *H. pylori* often induce diarrhea (Lopes et al., 2014; Majumdar & Looi, 2024).

The development prospects of this research are the study of the possible synergistic action of Ag- or Zn-clinoptilolite in combination with antibiotics used in therapies against *H. pylori*. The existence of a synergy could allow the reduction of the dose of metal and/or antibiotic necessary to kill the bacterium (Bu et al., 2024; Slavin et al., 2017; Yin et al., 2023), furthermore a formulation containing Ag(or Zn)-clinoptilolite + antibiotic could be effective against *H. pylori* strains resistant to antibiotics.

## Data Availability

No datasets were generated or analysed during the current study.
